# High Ionic Conduction in Rb‐ and Cs‐Mixed Cation Amide for Energy Storage

**DOI:** 10.1002/smll.202502943

**Published:** 2025-05-12

**Authors:** Thi Thu Le, Kai Sellschopp, Fabrizio Murgia, Anna Louise Garden, Simone Bordignon, Jan Peter Embs, Michele Remo Chierotti, Alexander Schökel, Fahim Karimi, Paul Jerabek, Thomas Klassen, Claudio Pistidda

**Affiliations:** ^1^ Institute of Hydrogen Technology Helmholtz‐Zentrum hereon GmbH D‐21502 Geesthacht Germany; ^2^ Department of Chemical Physical Mathematical and Natural Sciences University of Sassari Sassari 07100 Italy; ^3^ Department of Chemistry University of Otago Dunedin 9054 New Zealand; ^4^ The MacDiarmid Institute for Advanced Materials and Nanotechnology Victoria University of Wellington Wellington 6140 New Zealand; ^5^ Department of Chemistry and NIS Centre University of Torino Torino 10125 Italy; ^6^ PSI Center for Neutron and Muon Sciences PSI Villigen 5232 Switzerland; ^7^ Deutsches Elektronen‐Synchrotron DESY D‐22607 Hamburg Germany; ^8^ Helmut Schmidt University University of the Federal Armed Forces Hamburg D‐22043 Hamburg Germany

**Keywords:** amides, DFT, electrochemistry, ionic conductivity, ion exchange

## Abstract

Ionic conductivity is one of the key parameters in designing advanced solid‐state batteries and energy storage materials. This study presents the first observation of high ionic conductivity in the newly developed mixed cation amide solid solution, Rb_0.5_Cs_0.5_NH_2_, within the RbNH_2_‐CsNH_2_ system. In particular, the solid solution formed shows an unexpectedly high ionic conductivity that is four orders of magnitude higher than that of the individual compounds, RbNH_2_ and CsNH_2_. This substantial improvement is ascribed to the Rb^+^/Cs^+^ cation exchange process. This exchange significantly stabilizes the cubic structure, thereby enhancing ionic conductivity in the solid solution compared to the parent compounds. A combined experimental and computational study using quasielastic neutron scattering (QENS) and density functional theory (DFT) elucidates the mechanism of Rb^+^/Cs^+^ ion migration in solid solution. The findings indicate intrinsically correlated with the reorientation dynamics of [NH_2_]⁻ anions, which activates and facilitates Rb⁺/Cs⁺ ion transport within the lattice via the *paddlewheel mechanism*. A deep understanding of the crystal structure, anion reorientation dynamics, and cation migration mechanisms is crucial for advancing the ionic conductivity and hydrogen storage characteristics of these amide materials.

## Graphical abstract

A mixed cation‐amide solid solution, Rb_0.5_Cs_0.5_NH_2_, based on the interaction between RbNH_2_‐ and CsNH_2_, has been developed. The Rb⁺/Cs⁺ cationic substitution leads to structural stabilization, resulting in high ionic conductivity in the Rb_0.5_Cs_0.5_NH_2_ compared to the single compounds. The Rb⁺/Cs⁺ ion migration proceeds via the octahedral‐octahedral pathway and is facilitated by NH_2_^−^ reorientation via a *paddlewheel mechanism*.

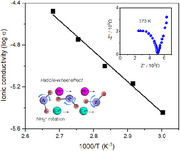

## 1 Introduction

The advancement of innovative energy storage materials is crucial for promoting sustainability and mitigating anthropogenic environmental impact. In this regard, complex hydrides have attracted considerable attention for their promising applications in hydrogen storage^[^ ^1^ ^]^ and as solid electrolytes.^[^ ^2^, ^3^, ^4^ ^]^ Experimental observations have shown that, for specific complex hydride‐containing systems such as LiNH_2_‐LiH,^[^ ^5^ ^]^ Mg(NH_2_)_2_‐LiH,^[^ ^6^ ^]^ Mg(NH_2_)_2_‐KH,^[^ ^7^ ^]^ and Mg(NH_2_)_2_‐RbH,^[^ ^8^ ^]^ fast (de)hydrogenation kinetics are achieved when high ionic conductive intermediate phases are formed. A prominent example is the LiNH_2_‐LiH system (referred to as Li‐N‐H). This system can reversibly release 6.5 wt.% hydrogen according to Equation (1), at temperatures below 573 K:

(1) $$\;LiNH_{2}+LiH⇄Li_{2}NH+H_{2}$$

Both LiNH_2_ and Li_2_NH exhibit a cubic close packed (*ccp*) anion structure. The structural transformation of LiNH_2_ and Li_2_NH proceeds through the formation of the Li_(1+x)_NH_(2–x)_ solid solution.^[^ ^5^ ^]^ This transformation is associated with the interdiffusion of Li^+^ into the LiNH_2_ cubic lattice and the concurrent expulsion of H^+^ ions. The room‐temperature (RT) ionic conductivity of Li_2_NH is 3 × 10^−4^ S⋅cm^−1^, which increases to 2 × 10^−2^ S⋅cm^−1^ at 373 K,^[^ ^9^ ^]^ while LiNH_2_ has an ionic conductivity of 10^−9^ S⋅cm^−1^ at RT and 5 × 10^−6^ S⋅cm^−1^ at 400 K.^[^ ^10^ ^]^ The low ionic conductivity in LiNH_2_ appears to be a kinetically limiting factor in the (de)hydrogenation of the pure Li‐N‐H system, and thus one of the causes of the sluggish (de)hydrogenation kinetics of the system. David et al.^[^ ^11^ ^]^ emphasized that the mobility of Li^+^ and H^+^ ions are key factors for the reversible hydrogenation (Equation (1)) or amide decomposition (Equation (2)) in the Li‐N‐H system.

(2) $$2LiNH_{2}\to Li_{2}NH+NH_{3}$$

The conversion mechanism between LiNH_2_ and Li_2_NH in both Equation (1) and Equation (2) are bulk reactions occurring through non‐stoichiometric processes driven by the migration of Li^+^ and H^+^ in the cubic lithium imide. The mechanism described in Ref. [11] is based on Frenkel defect pairs where Li^+^ ions diffuse through the cation vacancies. During this process, surface or migrating Li^+^ interacts with applied hydrogen molecules (H_2_) to form LiH and protonic hydrogen (H^+^). The H^+^ then interacts with the negatively charged [NH]^2−^ of Li_2_NH to form LiNH_2_, thus completing the hydrogenation (Equation (1)). Zhang *et al.* showed that Ti‐based catalysts such as TiCl_3_^[^ ^12^ ^]^ and LiTi_2_O_4_^[^ ^13^ ^]^ improve the hydrogen sorption kinetics in the Li‐N‐H system by facilitating Li⁺ and H⁺ transport at the LiH/LiNH_2_ interface, resulting in direct H_2_ formation during dehydrogenation. This catalytic doping increases the ionic conductivity by a factor of 1.5 and reduces the hydrogen desorption temperature and activation energy by ≈17%. Anderson et al.^[^ ^14^ ^]^ also found that higher Li⁺ conductivity correlates with faster (de)hydrogenation rates in LiNH_2_‐LiCl and Li_2_NH‐LiCl systems. Similarly, the addition of LiBH_4_ significantly improves the dehydrogenation kinetics, cyclability, and activation energy of the 6Mg(NH_2_)_2_‐9LiH, one of the most promising systems for hydrogen storage.^[^ ^6^ ^]^ LiBH_4_ appears to stabilize the dehydrogenated state (*i.e*., LiNH_2_) by forming Li_4_(BH_4_)(NH_2_)_3_. This phase is known to possess an ionic conductivity of 2 × 10^−4^ S⋅cm^−1^ at RT and 1 × 10^−3^ S⋅cm^−1^ at 373 K.^[^ ^3^, ^15^ ^]^ In this system, the formation of Li_4_(BH_4_)(NH_2_)_3_ was reported to promote the diffusion of ions across the interfaces of the amide‐hydride matrix.^[^ ^6^ ^]^ The results observed in these reports highlight the correlation between ionic conductivity and hydrogen‐sorption properties in complex hydride‐based materials and suggest that achieving high ionic mobility in the crystal lattice at interfaces will be essential for obtaining excellent hydrogen storage properties.

Recently, a series of mixed‐cation solid solutions formed by the interaction of amides and hydrides of the alkali metals K, Rb, and Cs was reported.^[^ ^8^, ^16^, ^17^ ^]^ At temperatures above 328 K, KNH_2_, RbNH_2_, and CsNH_2_ display comparable cubic structures (*space group, s.g*. $Fm\bar{3}m$) and form solid solutions with their corresponding hydrides, represented as M(NH_2_)_x_H_1−x_, through an interchange of NH_2_^−^/H^−^ anions within the MNH_2_‐MH systems (M = K, Rb, Cs). Furthermore, solid solutions of mixed metal amide hydrides are formed through both cationic and anionic substitution in the systems like KNH_2_‐RbH, RbNH_2_‐KH, RbNH_2_‐CsH, and CsNH_2_‐RbH. Therefore, we can assume that the increase of configurational entropy provided by the mixing of structurally similar reacting phases, as well as the lattice distortion originated by the different cations in the mixed amide, play a significant role in stabilizing, at lower temperatures, disordered polymorphs that showcase fast anionic reorientation, thus enhancing cation motion. As an example, in this study, we report on the unexpectedly high ionic conductivity of the Rb_0.5_Cs_0.5_NH_2_ system and elucidate its Rb^+^/Cs^+^ ion transport mechanism through a combination of experimental and computational techniques. Uncovering the related mechanisms of ion migration further provides general guidelines for improving the hydrogenation/dehydrogenation kinetics and the ionic conductivity in the class of complex amide‐hydride materials.

## 2 Results and Discussion

### 2.1 Formation of Rb_0.5_Cs_0.5_NH_2_ Solid Solution: Structural and Spectroscopic Insights

**Figure 1:**
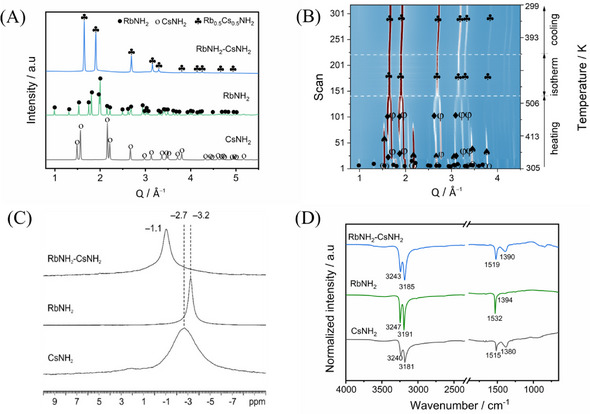
Structural and spectroscopic analysis: A) Ex situ XRD of CsNH_2_, RbNH_2_, and post‐heated mixed RbNH_2_‐CsNH_2_ samples. B) Phase evolution of the mixed RbNH_2_‐CsNH_2_ sample during the heating to 513 K, while in isothermal conditions for 10 min, and after cooling to RT. C) ^1^H MAS NMR spectra of the RbNH_2_, CsNH_2_ and post‐heated RbNH_2_‐CsNH_2_ samples. D) FT‐IR spectra acquired at RT for RbNH_2_, CsNH_2_, and post‐heated RbNH_2_‐CsNH_2_ samples.

Ex situ powder X‐ray diffraction (XRD) analysis, carried out at RT, shows significant structural changes for the pure compounds (RbNH_2_ and CsNH_2_) and their mixture after being heated to 513 K (**Figure** **1**A). Diffraction patterns of the pure compounds show the typical reflections of tetragonal CsNH_2_ (*s.g*. *P*4/*nmm*, denoted as ο) and monoclinic RbNH_2_ (*s.g*. *P*2_1_/*m*, denoted as •) phases, as previously reported.^[^ ^8^, ^16^ ^]^ In contrast, the mixed sample shows a single cubic phase (♣) indexed within *s.g*. $Fm\bar{3}m$, suggesting an interaction between RbNH_2_ and CsNH_2_ during heating. To elucidate the phase evolution during the formation of this cubic structure, synchrotron radiation powder X‐ray diffraction (SR‐PXD) was performed for the mixed sample under Ar atmosphere, with temperatures ramped up from RT to 513 K and then held isothermally for 30 min before cooling (Figure 1B). Initially, the monoclinic RbNH_2_ phase and tetragonal CsNH_2_ are observed. As the temperature increases, RbNH_2_ transforms to a cubic structure (φ) with *s.g*. $Fm\bar{3}m$ at ≈338 K, while CsNH_2_ initially converts to a cubic phase (♠) with *s.g*.$\;Pm\bar{3}m$ at ∼308 K, followed by a transition to cubic (♦) with *s.g*.$\;Fm\bar{3}m$ at ∼328 K. Above 510 K, the diffraction peaks of RbNH_2_ and CsNH_2_ merge and form a single solid solution phase, which remains stable during cooling. The Rietveld refinement analysis of the SR‐PXD pattern at RT reveals that the solid solution adopts a cubic structure, indexed within *s.g*.$Fm\bar{3}m$, with lattice parameter of *a* = 6.508554 (11) Å for the resulting Rb_0.5_Cs_0.5_NH_2_ (*s.g*. $Fm\bar{3}m$) phase (Figure S1, Supporting Information). This structure is similar to the high‐temperature polymorphs of CsNH_2_ and RbNH_2_ but has a different lattice parameter.

Direct evidence for solid‐solution formation is provided by solid‐state magic angle spinning nuclear magnetic resonance (^1^H MAS NMR) spectra at RT of both the initial and post‐heated mixed samples (Figure 1C). A single peak with similar chemical shift for the amide anion across all spectra suggests a similar local environment for the amide ions in both the pure materials and the mixed sample. The mixed sample shows a slightly higher chemical shift (−1.1 ppm), probably due to stronger hydrogen bonding. This is attributed to the substitution of Cs^+^ ion by the smaller Rb^+^ ion, together with the lower electronegativity of Cs compared to Rb, resulting in a bond‐strengthening contraction, which is further supported by the observed shifts in the N‐H vibrational modes in the solid solution (Figure 1D). Compared to CsNH_2_ alone, the stretching and bending vibrations of the N─H bond in the post‐heated RbNH_2_‐CsNH_2_ sample are shifted toward higher wavenumbers by 3–5 cm^−1^ and 5–10 cm^−1^, respectively.

### 2.2 Rb/Cs Ion Conduction and Transport Mechanism: Atomic Dynamics Investigations and DFT Calculations

**Figure 2:**
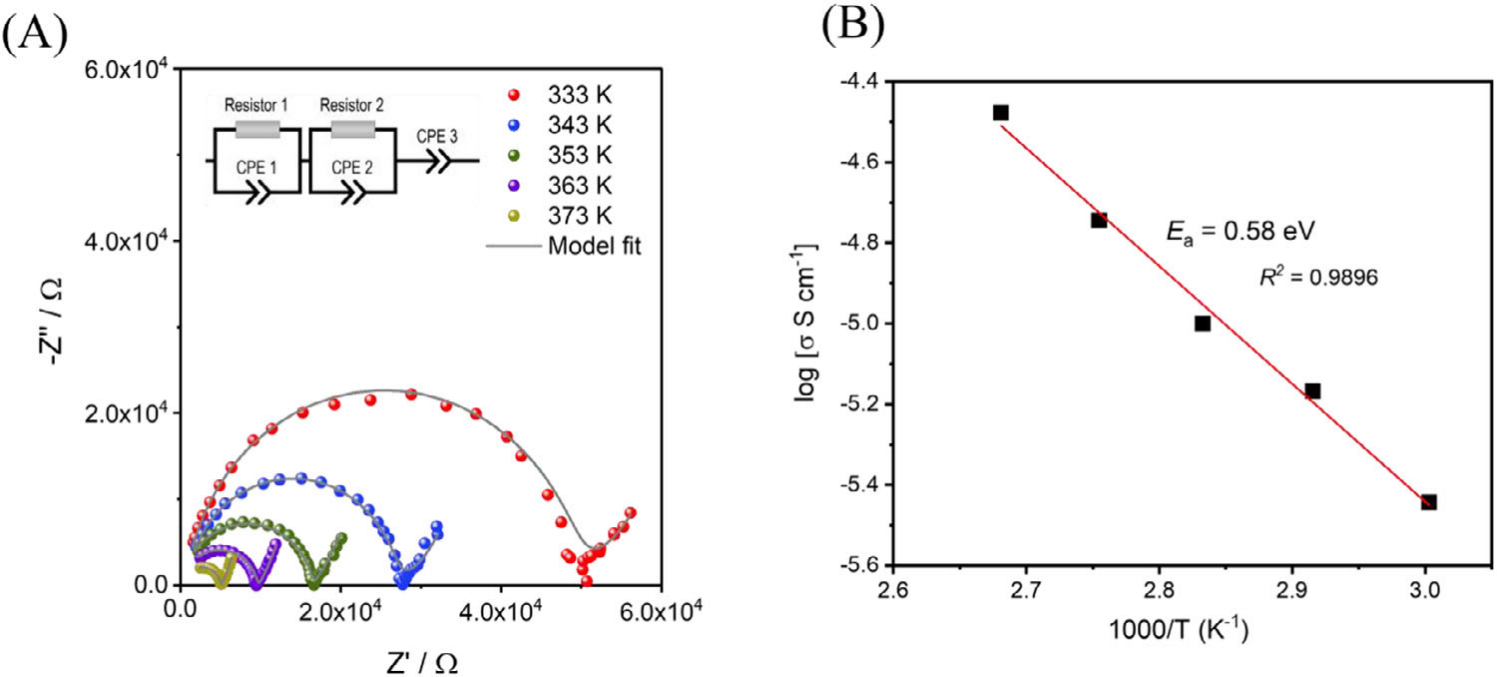
Impedance spectra analysis for the Rb_0.5_Cs_0.5_NH_2_ solid solution: A) Nyquist plots of the Rb_0.5_Cs_0.5_NH_2_ solid solution at different temperatures fitted with an equivalent circuit. B) Activation energy for Rb/Cs ion conduction obtained from the Arrhenius plot for temperature‐ dependent conductivity.

The Rb_0.5_Cs_0.5_NH_2_ solid solution exhibits significantly higher ionic conductivity compared to its individual component phases. The temperature‐dependent ionic conductivity of Rb_0.5_Cs_0.5_NH_2_ was measured by electrochemical impedance spectroscopy. The Nyquist plots with corresponding equivalent circuit fits, shown in **Figure** **2**A, were acquired over a temperature range of 333–373 K. It is noteworthy that an imperfect semicircle appears in the Nyquist plot even at 323 K (Figure S2A, Supporting Information), indicating an early onset of ionic conduction in the solid solution. The Nyquist spectra in Figure 2A show two distinct regions: a high‐frequency semicircle representing the real part of the impedance (Z′) corresponding to the bulk ionic conductivity, and a low‐frequency straight line resulting from electrode polarization. With increasing temperature, the semicircle shifts to lower impedance values, indicating an increase in ionic conductivity with temperature. All impedance data were fitted using the equivalent circuit (R_1_P_1_)‐(R_2_P_2_)‐P_3_, where R_1_ and R_2_ represent the bulk and interphase resistances, respectively, and P is the electrode contribution. At 333 K, the Rb_0.5_Cs_0.5_NH_2_ solid solution already exhibits a conductivity value of 4 × 10^−6^ S⋅cm^−1^, reaching 0.4 × 10^−4^ S⋅cm^−1^ at 373 K, whereas the pure materials showed mostly undetectable ionic conductivity at these temperature as the signal‐to‐noise ratio prevents a reasonable fit of the Nyquist plot (Figure S2B, Supporting Information).

The activation energy (*E_a_*) for ionic transport in the Rb_0.5_Cs_0.5_NH_2_ solid solution derived from an Arrhenius fit to the conductivity data was estimated to be 0.58 eV (Figure 2B), comparable to the activation energy for Li ionic conduction in high temperature (HT)‐LiBH_4_ (*E_a_* = 0.53 eV).^[^ ^18^ ^]^ The Rietveld refinement analysis of the SR‐PXD pattern for the Rb_0.5_Cs_0.5_NH_2_ solid solution (*s.g*. $Fm\bar{3}m$, *a* = 6.508554 Å) reveals that its structure resembles the high‐temperature polymorphs of CsNH_2_ (cubic, *s.g*. $Fm\bar{3}m$, *a* = 6.710440 Å) and RbNH_2_ (cubic, *s.g*. $Fm\bar{3}m$, *a* = 6.48491 Å), but with different lattice parameters. This suggests a stabilization of the cubic structure caused by the substitution of different cation size between Rb^+^ (r_ion_ = 1.61 Å) and Cs^+^ (r_ion_ = 1.74 Å), which provides a less densely packed structure and thus facilitates a more efficient Rb/Cs ion diffusion. Compared to the ionic conduction of smaller cations in amide compounds, the observed conductivity of this Rb_0.5_Cs_0.5_NH_2_ solid solution (0.4 × 10^−4^ S⋅cm^−1^ at 373 K) surpasses that of other alkali amide compounds. In KNH_2_, structural disorder facilities K^+^ ion transport, resulting in a conductivity of 3.56 × 10^−4^ S⋅cm^−1^ at 423 K.^[^ ^2^ ^]^ Similarly, cationic substitution of Li^+^ by Na^+^ in the LiNH_2_ results in an ionic conductivity on the order of 10^−5^ S⋅cm^−1^ at 375 K.^[^ ^19^ ^]^

**Figure 3:**
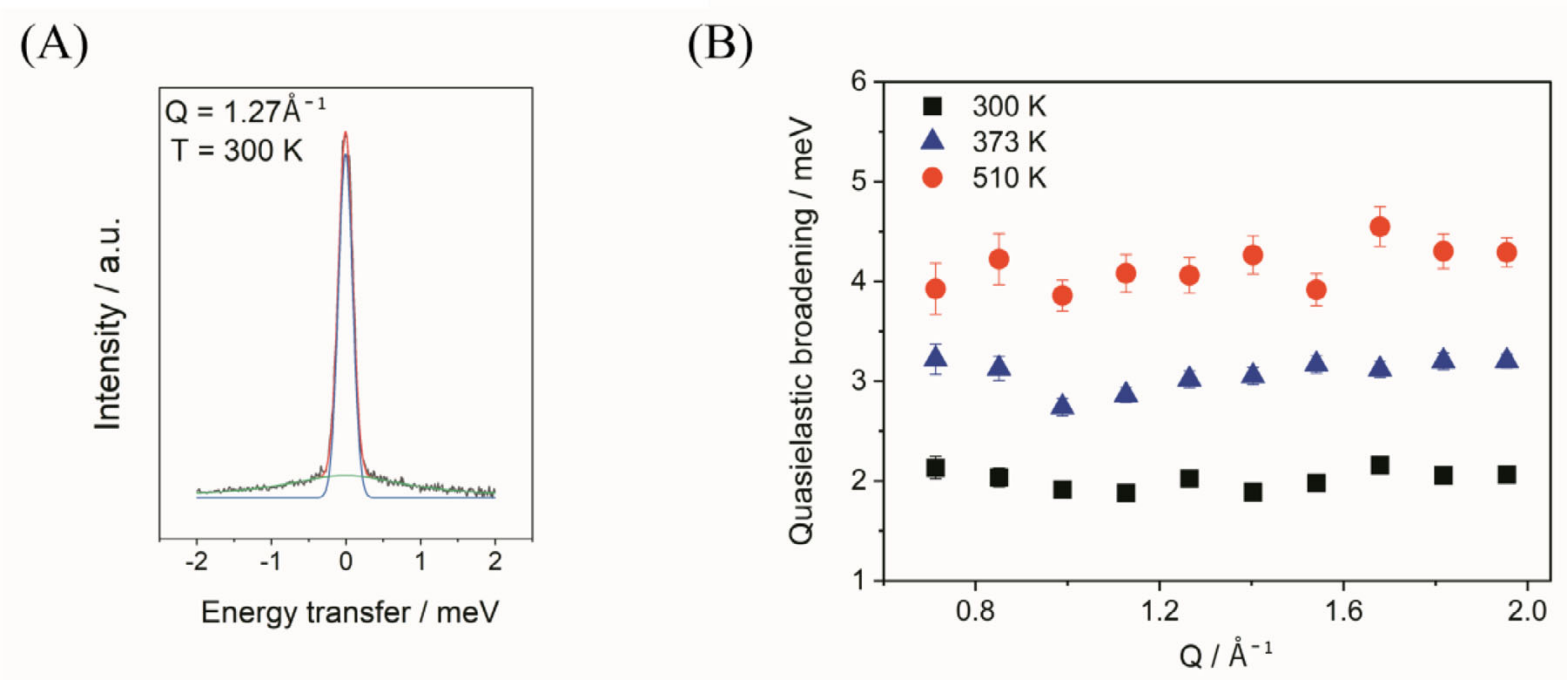
A) QENS spectrum of Rb_0.5_Cs_0.5_NH_2_ solid solution, measured at 300 K and at momentum transfer Q = 1.27 Å^−1^ using 4.4 Å incident neutrons. The black line displays the measured data. Red line represents fit to the data, with two components: *δ(ω)* function (blue line) and Lorentzian function (green line). B) Quasielastic broadening of the Rb_0.5_Cs_0.5_NH_2_ sample is obtained at different temperatures over a wide Q‐range from 0.7 to 2.09 Å^−1^.

To understand the increased cation mobility, we investigated the dynamics of the NH_2_^−^ anion in the Rb_0.5_Cs_0.5_NH_2_ solid solution using QENS). The QENS spectra were fitted using Equation (4), which includes an elastic term represented by the Delta function *δ(ω)* and a quasielastic part described by a Lorentzian function *L(Q,ω)*. An example of the QENS fit at 300 K with a momentum transfer of 1.27 Å^−1^ is given in **Figure** **3**A. The quasielastic broadening increases with temperature (Figure 3B), indicating accelerated motion at elevated temperatures.

**Figure 4:**
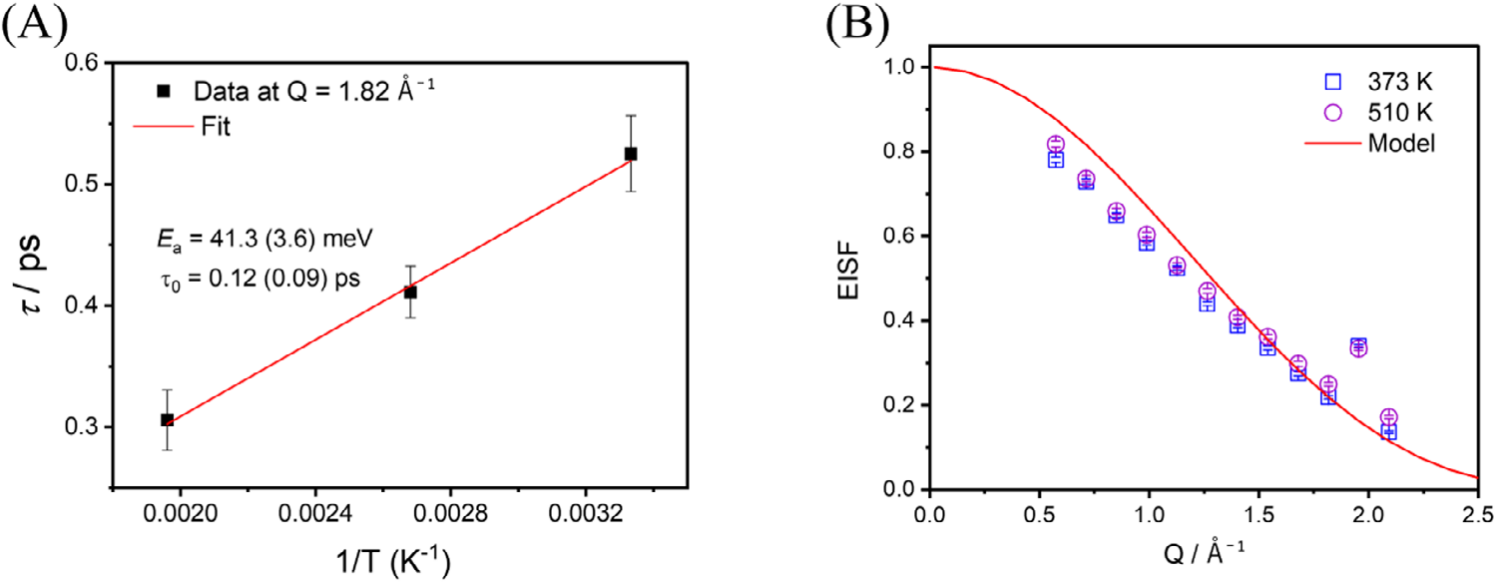
Dynamics of NH_2_ group in the Rb_0.5_Cs_0.5_NH_2_ solid solution observed by QENS: A) Thermally activated Arrhenius behavior of the NH_2_ rotational motion. B) Q‐dependent EISF of the Rb_0.5_Cs_0.5_NH_2_ solid solution at 373 and 510 K, fitted with model EISF describing 90° reorientations of the NH_2_^−^ anion around *C*_4_ axes according to Equation (3).

The stepwise NH_2_^−^ rotations in the Rb_0.5_Cs_0.5_NH_2_ solid solution follow the Arrhenius law: $\tau =\tau_{0}\exp\left(\frac{E_{a}}{k_{B}T}\right)$, where τ is the mean residence time for each successive rotational step derived from the Lorentzian FWHM, τ  ∼  2ℏ/*FWHM*. **Figure** **4**A shows the Arrhenius plot of τ versus temperature, yielding an activation energy *E_a_* ∼ 41.3(3.6) meV and an attempt frequency *τ_0_* ^−1^ on the order of 10^−12^ s^−1^. The activation energy of the NH_2_^−^ reorientations in the Rb_0.5_Cs_0.5_NH_2_ solid solution is rather low, which is comparable to that for the NH_2_^−^ rotation in KNH_2_ and close to the energies of liberational modes. The reorientation behavior of the NH_2_^−^ ion in this solid solution can be captured by the elastic incoherent structure factor (EISF). Figure 4B shows the EISF obtained from the QENS data fitted with a cubic‐phase model describing 90° NH_2_ reorientations ^20^ around the fourfold axes according to Equation (3):

(3) $$EISF\left(Q\right)=\frac{1}{8}\left[1+3j_{0}\left(Qa\right)+3j_{0}\left(\sqrt{2}Qa\right)+j_{0}\left(\sqrt{3}Qa\right)\right]$$

where j_0_(x) = sin (x)/x represents the zeroth‐order spherical Bessel function, *a* is the length of the cubic edges where the equilibrium hydrogen positions are located. As shown in Figure 4B, the modeled EISF for 90° reorientations of the NH_2_^−^ ion is in good agreement with the measured data for the higher Q values (≥1.0 Å^−1^). However, for Q < 1.0 Å^−1^, the model shows discrepancies, probably due to multiple scattering effects at small scattering angles. In addition, the data point at Q ∼ 1.92 Å^−1^ clearly deviates from the fit of the neighboring data points, likely due to the presence of a Bragg peak near this Q position. Therefore, it is most likely that the NH_2_^−^ anions in the Rb_0.5_Cs_0.5_NH_2_ solid solution samples rotate 90° around the *C*_4_ axes, similar to the NH_2_^−^ reorientational behavior of NH_2_^−^ anion observed in RbNH_2_ (Figure S3A, Supporting Information) and CsNH_2_ (Figure S3B, Supporting Information), in KNH_2_,^[^ ^20^ ^]^ and the BH_4_^−^ ion in the alkaline tetrahydroborides such as HT‐LiBH_4_, NaBH_4_, and KBH_4_.^[^ ^21^ ^]^ Fitting Equation (3) to the measured EISF data yields the cubic edge length *a* = 1.28 (*0.2*) Å for the equilibrium positions of the hydrogen atom (H‐H distance) at the corners during reorientation, with a radius *R* = 1.10 (*0.1*) Å for the sphere containing that cube. This value is slightly larger than the expected N‐H bond distance (1.03 Å),^[^ ^22^ ^]^ suggesting that the NH_2_^−^ reorientation is not purely rotational but also coupled to a translational motion, probably involving the center‐of‐mass motion of the cation. This effective spatial range over which the hydrogen atoms move during reorientation defines a geometric threshold for the mobility of the cation, for which smaller values can hinder translational motion, leading to a higher energy barrier for cation transport, as further confirmed by the subsequent DFT calculations with the NH_2_^−^ anions are fixed (see Figure S4, Supporting Information).

**Figure 5:**
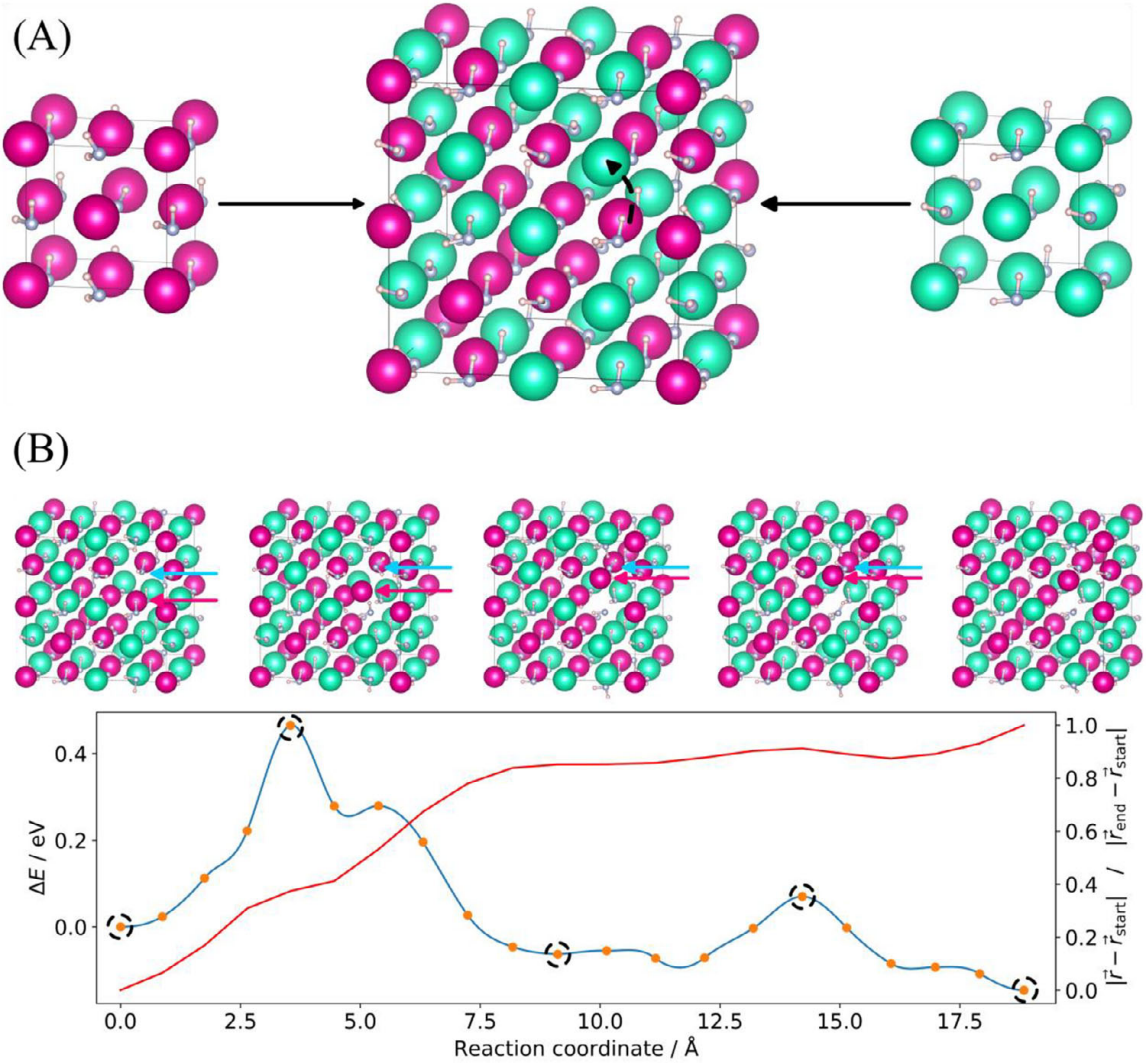
Energy barriers for ion motion in Rb_0.5_Cs_0.5_NH_2_ calculated by DFT: A) Calculated structures of RbNH_2_ (left), CsNH_2_ (right), and the SQS for modeling the Rb_0.5_Cs_0.5_NH_2_ solid solution (center). The studied migration path for the barrier study is marked with a dashed black arrow. Color code: Rb – magenta, Cs – aqua, N – blue, H – white. B) Calculated minimum energy path for the RbNH_2_‐CsNH_2_ solid solution. Top: Atomic structures of selected images along the paths (marked by dashed circles), color code: Rb – magenta, Cs – aqua, N – blue, H – white, moving cation marked with magenta arrow, anion closest to the path marked with blue arrow. Bottom: Energy profile of the path (blue line with orange circles) and relative distance of the cation to the starting position (red line).

To further understand the ion transport mechanisms in the Rb_0.5_Cs_0.5_NH_2_ solid solution, we performed DFT calculations. Using a special quasi‐random structure (SQS) model optimized for volume and atomic positions of the NH_2_^−^ anions, we generated a 128‐atom configuration and applied the climbing image nudged elastic band (CI‐NEB) method to identify and optimize a cation migration path, as highlighted in **Figure** **5**A. The resulting minimum energy path is shown in Figure 5B, along with key intermediate states. An animation of the atomic structure evolution along the path is provided in the Movie S1 (Supporting Information). The computed activation energy of 0.47 eV is reasonably consistent with the experimental value of 0.58 eV, considering known simulation‐experiment discrepancies and the fact that vibrational effects are not included in the model. Figure 5B further illustrates the relative movement of the cation along the path, with most of the displacement occurring early in the path. Notably, a smaller barrier of 0.13 eV in the second part is not associated with significant cation movement. It should be noted that the cationic movement is different from the reaction coordinate, which is usually used as the horizontal axis in such plots and integrates *all* atomic rearrangements in the structure. Examination of the atomic structures along the migration path reveals significant rearrangements of the NH_2_^−^ anions, particularly those closest to the migrating cation. In the initial phase of the minimum energy path, cation transport is coupled with two 90° rotations of the closest NH_2_^−^ anion as well as additional 90° rotations of several surrounding NH_2_^−^ anions. In the latter part of the path, the most significant atomic rearrangement is a 90° rotation of the closest NH_2_^−^ anion, corresponding to a lower energy barrier of 0.13 eV. This observation is consistent with the EISF model derived from QENS data. This coordination between cation movement and rotational reorientations of neighboring ions is consistent with the physically motivated definition by Smith and Siegel of the so‐called “*paddlewheel mechanism*”,^[^ ^23^ ^]^ which is assumed to facilitate ionic conduction in a variety of ionic conductors.^[^ ^24^, ^25^ ^]^ The key aspects for the presence of a *paddlewheel effect* according to Siegel and Smith are: i) large anion rotations that facilitate cation movement while maintaining local coordination and ii) temporal and spatial correlation of anion reorientations and cation hopping at comparable rates. From our point of view, these characteristics are met for the described and visualized reaction pathway (see Movie S1, Supporting Information, time stamp 0:02–0:06, bottom‐right view), where it is clearly visible how the NH_2_^−^ anion rotates counter‐clockwise to allow transition of Rb^+^/Cs^+^. The correlation between anionic reorientation and cation motion has also been recently proved for other Cs‐containing crystal structures featuring large cage anions, i.e. CsCB_11_H_12_. QENS measurements and ab initio calculations confirm this correlation, especially when thermal energy is sufficient to induce structural transitions toward disordered polymorphs.^[^ ^26^ ^]^ While the effect on the Cs^+^ mobility is less pronounced due to its large ionic radius, these findings are consistent with observations in analogous salts containing smaller cations.^[^ ^27^ ^]^ However, we are aware that the *paddlewheel effect* has been the subject of considerable debate in the field of solid‐state ionics,^[^ ^23^, ^28^, ^29^, ^30^, ^31^, ^32^ ^]^ and we have therefore included a more detailed discussion of this topic in the Supporting Information.

In addition, to assess the influence of the bottleneck size on the cation mobility induced by the NH_2_^−^ reorientation, we examined the effect of constraining the anion motion in our DFT calculations. Fixing the NH_2_^−^ anions resulted in a significant energy difference of ≈1.9 eV between the initial and final states of the NEB pathway, due to the suppressed anion reorientation toward their energetically favorable configurations. Likewise, the energy barrier for cation migration is significantly increased to ∼ 2.3 eV under these conditions (see Figure S4, Supporting Information), highlighting the importance of coupled cation‐anion dynamics.

Visualization of the *fcc* crystal structure of Rb_0.5_Cs_0.5_NH_2_ shows that Rb^+^ and Cs^+^ ions occupy octahedral sites, with the edges of these octahedra determined by the distances between neighboring NH_2_^−^ groups (Figure S5A, Supporting Information). Due to limitations in X‐ray diffraction, the exact position of the hydrogen atoms in the crystal structure cannot be directly observed; however, based on similar compounds, the N─H bond length is estimated to be ≈1.03 Å,^[^ ^22^, ^33^ ^]^ while Rietveld refinement gives an N‐N distance of 4.78 Å. Therefore, the length of an octahedral edge is ≈2.72 Å. Moreover, the arrangement of NH_2_^−^ units create unoccupied tetrahedral sites, which share triangular faces with the octahedra (see Figure S5A, Supporting Information). However, these sites are too small for the large cations (ionic radii Rb^+^ = 1.61 Å, Cs^+^ = 1.74 Å) to migrate to other octahedral sites. This observation is further confirmed by our DFT calculations, which indicate that in all cases the transition states are ≈1 Å away from the tetrahedral site, but never at this site. When a cation is placed at a tetrahedral site and the structure relaxes, the cation returns to the octahedral site, suggesting that the tetrahedral site is unstable. While the instability of the tetrahedral sites in the DFT calculations could be related to the *fcc* symmetry constraint for the remaining cations, it is also consistent with the XRD data showing no occupancy of tetrahedral sites. We therefore conclude that the Rb⁺ and Cs⁺ migration occurs directly along the octahedral‐octahedral (O‐O) pathway, rather than the octahedral‐tetrahedral‐octahedral (O‐T‐O) pathway observed for the conduction of smaller cations, such as Li, in other *fcc*‐structured sulfides and oxides.^[^ ^34^, ^35^, ^36^ ^]^ As shown in Movie S1 (Supporting Information), the calculated migration path of the cation follows the O‐O path in the y‐z plane (bottom left), passing through the edges of the octahedra which are not favorable for migration. As observed, the cation also moves in the x‐y (top left) and x‐z (top right) planes to avoid the direct path through the octahedral edges. However, it does not completely move to the tetrahedral site as this would result in it moving too close to other ions. In addition, since the DFT calculated energy barriers for ionic conduction in the pure systems in their cubic structure, 0.42 eV for RbNH_2_ and 0.52 eV for CsNH_2_ (Figure S5B,C, Supporting Information), are similar to the one in the Rb_0.5_Cs_0.5_NH_2_ solid solution system, we attribute the enhanced ionic conductivity of the solid solution to the stabilization of the cubic structure rather than the change in chemistry.

## 3 Conclusion 

This study demonstrates the formation of the Rb_0.5_Cs_0.5_NH_2_ solid solution, which exhibits a conductivity of ≈0.4 × 10^−4^ S·cm⁻¹ at 373 K and a low activation energy of 0.58 eV, within the RbNH_2_‐CsNH_2_ system. The improved performance is attributed to the stabilization of a cubic structure of the solid solution induced by the cation substitution, which significantly increases the ionic conductivity relative to the individual components. In addition, the excellent agreement between experimental results and computational predictions indicates the Rb⁺/Cs⁺ ion migration via the O‐O pathway and confirms that the fast ionic transport in Rb_0.5_Cs_0.5_NH_2_ is facilitated by the reorientation of NH₂ ions, suggesting a *paddlewheel mechanism* of ionic conduction similar to that observed in systems containing smaller cations such as Li and Na. These findings provide a basis for further studies on ionic conductivity and ion transport mechanisms in complex amide‐hydrides, which are being explored as hydrogen storage materials and solid electrolytes.

## 4 Experimental Section/Methods

### Materials Preparation

Rubidium amide (RbNH_2_) and cesium amide (CsNH_2_) were prepared following the procedures reported in references^[^ ^8^, ^16^ ^]^ The mixed sample was prepared by mixing RbNH_2_ and CsNH_2_ in a molar ratio of 1:1; the mixture was then heated from RT to 513 K and kept it in isothermal conditions for 3 h in Ar atmosphere. To avoid detrimental atmospheric contaminations, all samples were prepared in an Ar‐filled glovebox (MBraun, Germany) with oxygen and moisture concentration  ≤ 0.1 ppm.

### Materials Characterization

Ex situ powder X‐ray diffraction (XRD) experiments were performed using a D8 Discover diffractometer (Bruker AXS GmbH, Karlsruhe, Germany) equipped with a Cu K*α* radiation (λ = 1.54184 Å) and 2D VANTEC detector. The diffractograms were acquired in the 2*θ* range from 10° to 90°, in nine steps each of 8.9° with an exposure time of 300 s per step. All material handling was carried out in an Ar‐filled glovebox (oxygen and moisture concentration ≤0.1 ppm). A small amount of powder sample was placed into an airtight sample holder made of poly(methylmethacrylate) and then transported to the diffractometer.

In situ synchrotron radiation powder X‐ray diffraction (in situ SR‐PXD) experiments were carried out at the powder diffraction and total scattering beamline (P02.1) in Petra III (Desy Hamburg, Germany)^[^ ^37^ ^]^ using a monochromatic X‐ray beam (wavelength λ = 0.20734 Å). The diffraction patterns were collected using a Varex4343CT detector with an array of 2880 × 2880 pixels and a pixel size of 150 µm × 150 µm, using an exposure time of 10 s per scan. The samples were loaded under a purified Ar atmosphere in sapphire capillaries and then mounted on an in‐house developed in situ cell, in which both operating temperatures and pressures are precisely controlled.^[^ ^38^, ^39^ ^]^ All measurements were carried out under 1 bar of Ar. The sample were heated up from RT to 513 K with a heating rate of 10 K⋅min^−1^, then kept at 513 K for 30 min, and finally cooled to RT. The obtained 2D diffraction images were integrated into 1D diffractograms using the FIT2D software, quantitative analyses were performed by the Rietveld refinement method using the MAUD software.^[^ ^40^ ^]^ Structural information of known phases was obtained from the International Crystal Structure Database (ICSD).

Solid‐state nuclear magnetic resonance (SSNMR) experiments were run on a Jeol ECZR 600 instrument, operating at a frequency of 600.13 MHz for ^1^H and equipped with a 3.2 mm probe. Rotors were packed inside a glovebox to prevent sample decomposition. The ^1^H MAS spectra were acquired at probe temperature at a spinning speed of 20 kHz (4 scans; optimized relaxation delays equal to 200 or 280 s, corresponding to 5⋅T_1_ for quantitative measurements). The ^1^H chemical shift scale was calibrated with adamantane (^1^H signal at 1.87 ppm with respect to primary standard tetramethylsilane) as an external standard.

### Fourier‐Transform Infrared Spectroscopy (FT‐IR)

The pure and mixed samples were characterized using Fourier‐transform infrared spectroscopy (Cary 630 FT‐IR spectrometer, Agilent Technologies Deutschland GmbH, Waldbronn, Germany). The FT‐IR spectrometer was placed in an Ar‐circulated glovebox with oxygen and moisture concentrations below 5 ppm. The background was calibrated for each measurement; a small amount of material was placed on the diamond ATR top plate, and the FT‐IR spectrum was acquired at RT in a full frequency range of 4000–650 cm^−1^ with a spectral resolution of 4 cm^−1^ and the number of scans 300.

Quasielastic neutron scattering (QENS) measurements were conducted with cold neutrons using the time‐of‐flight neutron spectrometer (FOCUS) at the continuous spallation source SINQ, located at the Paul Scherrer Institute (Villigen, Switzerland). The incident neutron wavelength was 4.4 Å, and the elastic scattering energy resolution, *δ(E)*, was 0.229 meV (full width at half maximum, FWHM), corresponding to an observable time *τ* ≈ 2ℏ/FWHM of 5.7 ps. The samples were loaded into sealed aluminum cylinders (6‐mm diameter) inside the continuously Ar‐filled glovebox. The spectra were recorded over a range of scattering vectors from Q = 0.57 to 2.09 Å^−1^, with the length scale of the measurement depending on the Q range, the smallest measured Q provides the largest probed distance (*d*). Data reduction was performed using “DAVE” software (data analysis and visualization environment).^[^ ^41^ ^]^ The acquired QENS spectra were analyzed using the curve fitting utility “PAN” included in DAVE. The measured total incoherent scattering function *S(Q,ω)* can be expressed as a convolution of an elastic scattering described by the delta function *δ(ω)* and a quasielastic contribution described by the Lorentzian function *L(Q,ω)*, with a flat background (*bg*) to account for fast motions and the resolution function of the instrument *R(Q,ω)* determined using a vanadium standard:

(4) $$S{\left(Q,\omega \right)}_{\exp}=R\left(Q,\omega \right)⊗\left[I_{\mathrm{el}}\delta \left(\omega \right)+I_{\mathrm{qe}}L\left(Q,\omega \right)\right]+\mathrm{bg}$$

where *I_el_* and *I_qe_* are the integrated elastic and quasielastic scattering intensities, respectively. In addition, each component has its own width, *i.e*., the widths (HWHM) of Lorentzian functions, *Γ* ≈ ℏ/*τ*, are related to characteristic time scales of the motions being probed, which increase with increasing temperatures. If the elastic and quasielastic contributions are separated, the elastic incoherent structural factor can be calculated from the measured QENS data, as follows:

(5) $$EISF\;\left(Q\right)=A_{0}\;\left(Q\right)=\frac{I_{el}\left(Q\right)}{I_{el}\left(Q\right)+I_{qe}\left(Q\right)}$$

Electrochemical impedance spectroscopy (EIS): the ionic conductivity of materials was measured using a potentiostatic impedance analyzer (PSTAT30 potentiostats, Autolab, Germany). Powder samples were pressed into pellets with a thickness of 0.8–1.0 mm and a diameter of 10 mm using an axial hydraulic press with a pressure of 2–3 tons, the sample pellets were sandwiched between Indium foils (Alfa Aesar 0.125 mm thick, 99.99%) to improve contact with the electrodes and mounted in an airtight cell with stainless‐steel electrodes (rhd TSC battery cell). Impedance measurements were performed in the range of 300 K to 373 at 10 K intervals, and Nyquist plots were recorded in the frequency range of 1 MHz to 0.1 Hz and an AC perturbation of 10 mV. Impedance spectra was analyzed with the RelaxIS3 software from the rhd Instruments. The ionic conductivity (σ) was then calculated using the following equation $\sigma =\frac{1}{R\;}\times \frac{d}{A}$, where *R* (Ω‐Ohm) is the electrical impedance, determined from the fitted diameter of a single arc and the intercept of the Z’ axis in the Nyquist plot, *d* is the thickness of the pellet sample (cm), and *A* is the active contact area of the sample with the electrodes (cm^2^). The activation energy for ionic conduction (*E_a_*) is the slope of the ln (σ) vs.1/*T* plot of the Arrhenius equation:

(6) $$\sigma =\sigma_{0}\exp\left(-\frac{E_{A}}{RT}\right)$$

### Computational Methods

Density functional theory (DFT) calculations were carried out with VASP (version 6.3.0)^[^ ^42^, ^43^ ^]^ to study the diffusion barriers and underlying atomic processes in the pure and mixed amide systems. As for a previous study on similar systems,^[^ ^16^ ^]^ the GGA xc‐functional PBEsol^[^ ^44^ ^]^ was selected and corrected for dispersive interactions through the DFT‐D3 correction with Becke‐Johnson damping.^[^ ^45^, ^46^ ^]^ The PAW formalism implemented in VASP combined with the provided pseudopotentials (version 54) allowed to only treat the outer electrons of each element explicitly, which are the electrons from the 1s^1^ orbitals for hydrogen, the 2s^2^2p^3^ orbitals for nitrogen, the 4s^2^4p^6^5s^1^ orbitals for rubidium, and the 5s^2^5p^6^6s^1^ orbitals for cesium. Furthermore, an energy cut‐off of 500 eV, a Gamma‐centered k‐point grid with a maximum spacing of 0.5 Å^−1^, and a Gaussian smearing with a width of 0.02 eV were employed to reach energy convergence within less than 1 meV atom^−1^. The initial cubic crystal structures of RbNH_2_ and CsNH_2_ were obtained from the optimization procedure described in the previous work. ^[^ ^16^ ^]^ Additionally, the volumes of the structures were optimized through a Birch‐Murnaghan fit to the calculated energy‐volume curve. In each step of the volume optimization and for the final minimum‐energy structures, the face‐centered cubic (*fcc*) structure of the cations found in experiments was enforced during the calculation by fixing the unit cell and the position of all cations (Rb^+^, Cs^+^), while relaxing the nitrogen and hydrogen atoms until their forces were below 0.005 eV Å^−1^. The solid solution structure of the mixed amide is approximated by a special quasi‐random structure (SQS)^[^ ^47^ ^]^ that was created with the help of icet.^[^ ^48^ ^]^ Starting from the optimized RbNH_2_ and CsNH_2_ structures, the lattice constant was averaged and a 2 × 2 × 2 supercell was generated as an initial guess for the solid‐solution structure. Then, the site occupancies of the cation sites were modified randomly in a Monte Carlo‐based simulated annealing simulation (10^7^ steps) under the condition of keeping a 1:1 ratio between Rb and Cs to find an SQS with a cluster vector that matches the ideal random mixture of an infinite system as close as possible. The SQS was then optimized with the same procedure as for the pure amide structures. Diffusion barriers were calculated with the climbing image nudged‐elastic band (CI‐NEB) method as implemented in the VASP Transition State Tools (VTST).^[^ ^49^ ^]^ Each diffusion path was modeled by first removing a cation at the initial and final site, creating the final and initial structures, respectively, then relaxing the moving cation and all N and H atoms closest to the path until all forces were below 0.005 eV Å^−1^, and finally optimizing the path with the NEB method until the NEB forces were below 0.01 eV Å^−1^. Since removing a cation leaves behind a negative charge, all diffusion path calculations were performed on charged systems and charged neutralized by a homogeneous background charge, and a corresponding monopole correction was applied based on the computed dielectric constants of the non‐defective systems.^[^ ^50^ ^]^ A linear interpolation between the relaxed initial and final structures with 9 images was employed as the initial guess of the diffusion path. Whenever a significant additional energy minimum was found along the path, it was split up into sub‐paths that were individually converged using the method described above. As a final step, the climbing‐image method was applied to each (sub‐)path to determine the transition state with higher accuracy.

### Ethical Statement

This research follows the core values of Helmholtz‐Zentrum Hereon and adheres to transparency, integrity, and accountability in every aspect of the work.

## Conflict of Interest

The authors declare no conflict of interest.

## Author Contributions

T.T.L. wrote the original draft, performed conceptualization, methodology, validation, formal analysis, and investigation. K.S. performed methodology, validation, and investigation. F.M. performed the methodology and validation. A.L.G. performed the methodology. S.B. performed the methodology and investigation. J.P.E. acquired resources. M.R.C. performed the methodology and investigation. A.S. acquired resources. F.K. performed the investigation. P.J. contributed to validation T.K. performed funding acquisition and project administration. C.P. contributed to the writing of the original draft, and performed project administration and supervision. All authors contributed to the review and editing of this paper.

## Supporting information



Supporting Information

Supplemental Movie 1

## Data Availability

The data that support the findings of this study are available from the corresponding author upon reasonable request.
