# Supplementary material for: High Ionic Conduction in Rb‐ and Cs‐Mixed Cation Amide for Energy Storage
Source: Small. 2025 May 12;21(36):2502943. doi: 10.1002/smll.202502943 (PMC12423921; doi:10.1002/smll.202502943)
Supplement: Supplementary file 1 — Supporting Information [file SMLL-21-2502943-s002.docx]

Supporting Information

High Ionic Conduction in Rb- and Cs-Mixed Cation Amide for Energy Storage

Thi Thu Le*, Kai Sellschopp, Fabrizio Murgia, Anna L. Garden, Simone Bordignon, Jan Peter Embs, Michele R. Chierotti, Alexander Schökel, Fahim Karimi, Paul Jerabek, Thomas Klassen, Claudio Pistidda*


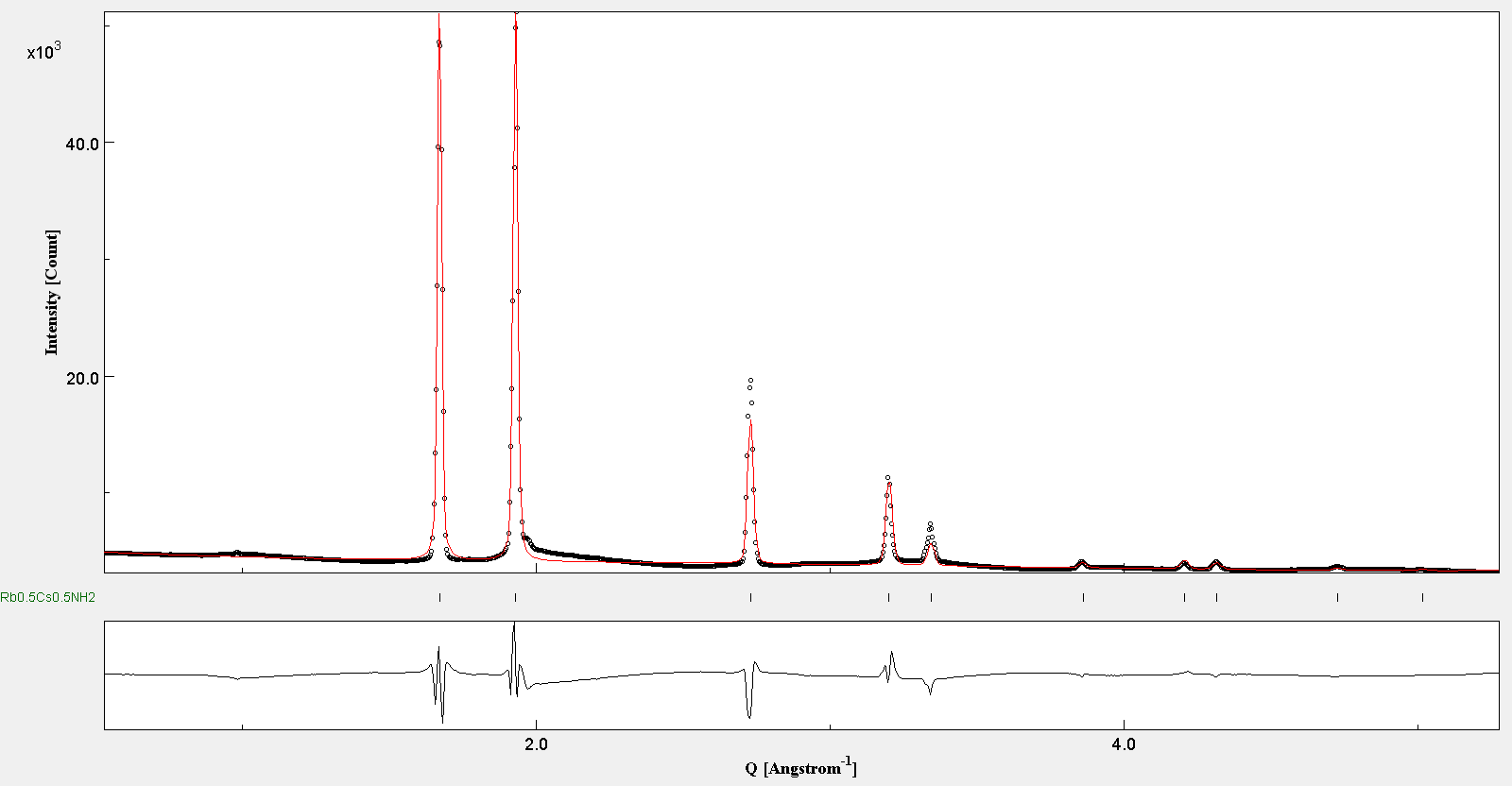


**Figure S1.** Rietveld refinement of the RT in situ SR-PXD data for the RbNH_2_-CsNH_2_ solid solution after being heated to 513 K. A structure for Rb_0.5_Cs_0.5_NH_2_ was indexed within *s.g*.$Fm\bar{3}m$ with lattice parameter of *a* = 6.508554 (11) Å. *R_wp_* = 6.5 %.


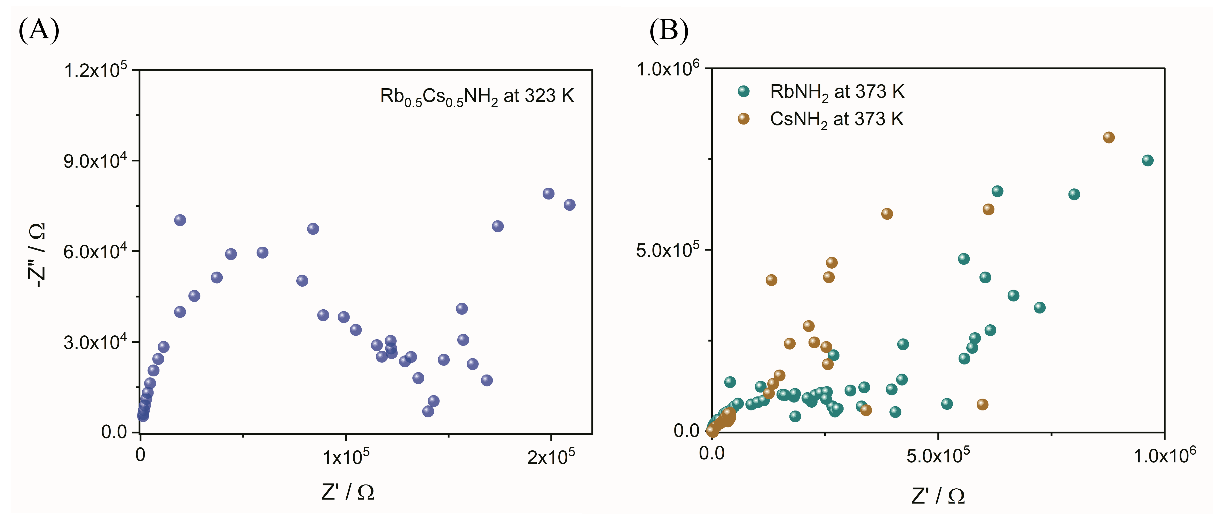


**Figure S2**. (**A**) Nyquist plots of RbNH_2_ and CsNH_2_ at 373 K. (**B**) Nyquist plot of Rb_0.5_Cs_0.5_NH_2_ at 323 K.


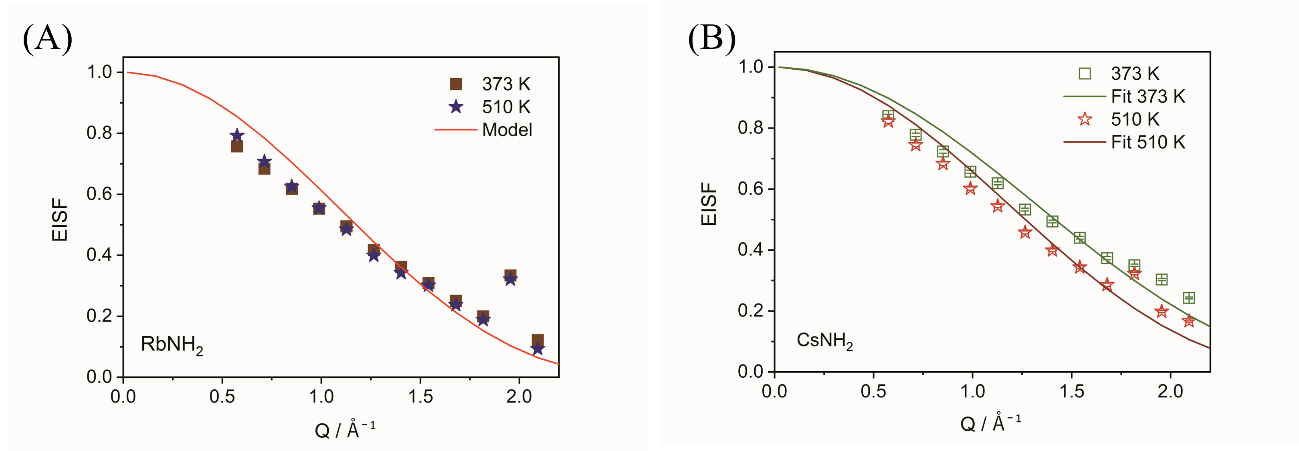


**Figure S3**. Measured EISF fitted with EISF model describing 90° reorientations of the NH_2_ ion in (A) RbNH_2_ and (B) CsNH_2_.

**
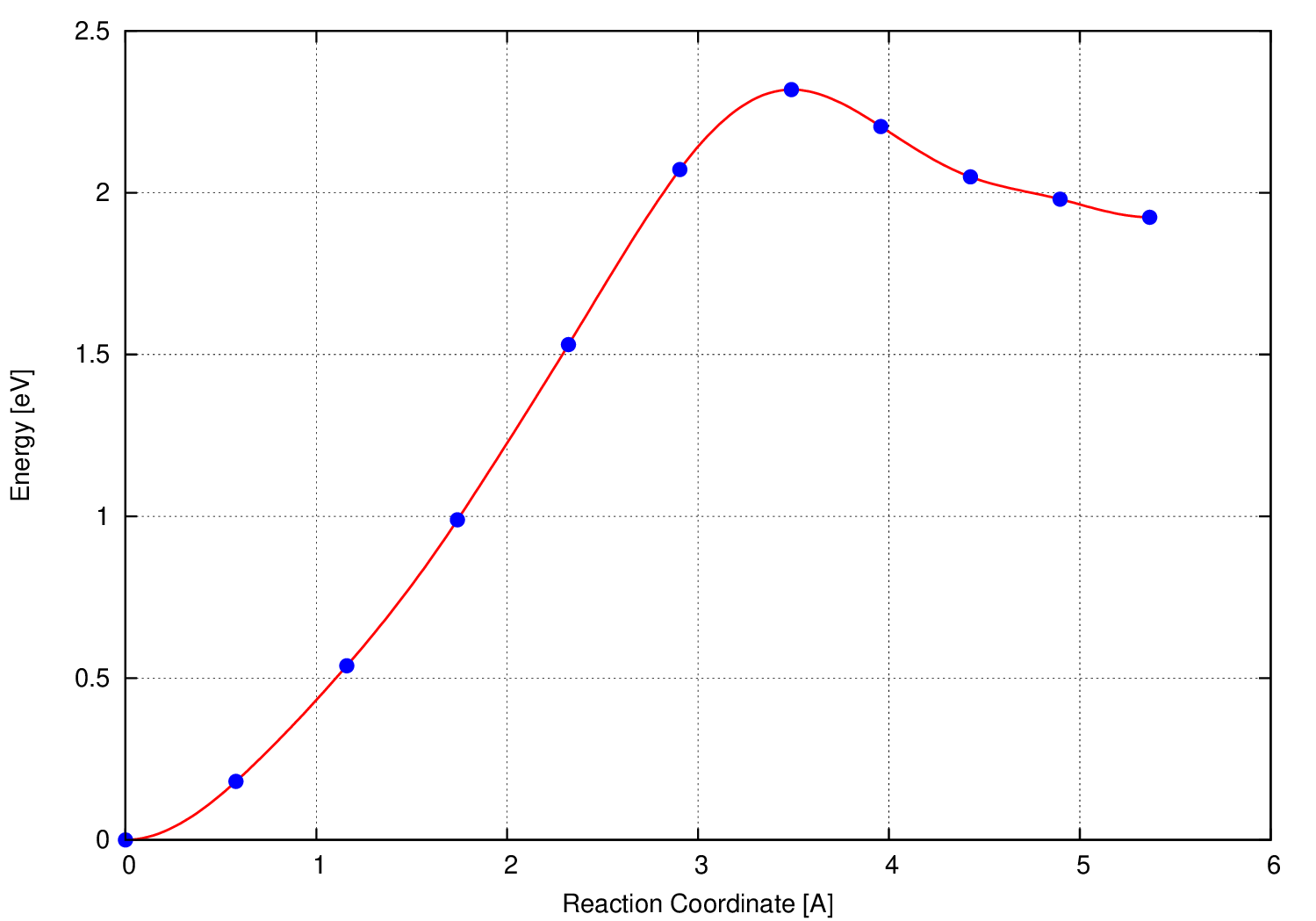
**

**Figure S4**. Cation migration energy profile along the O-O path in the Rb_0.5_Cs_0.5_NH_2_ when all NH_2_^–^ anions are fixed in their initial position.


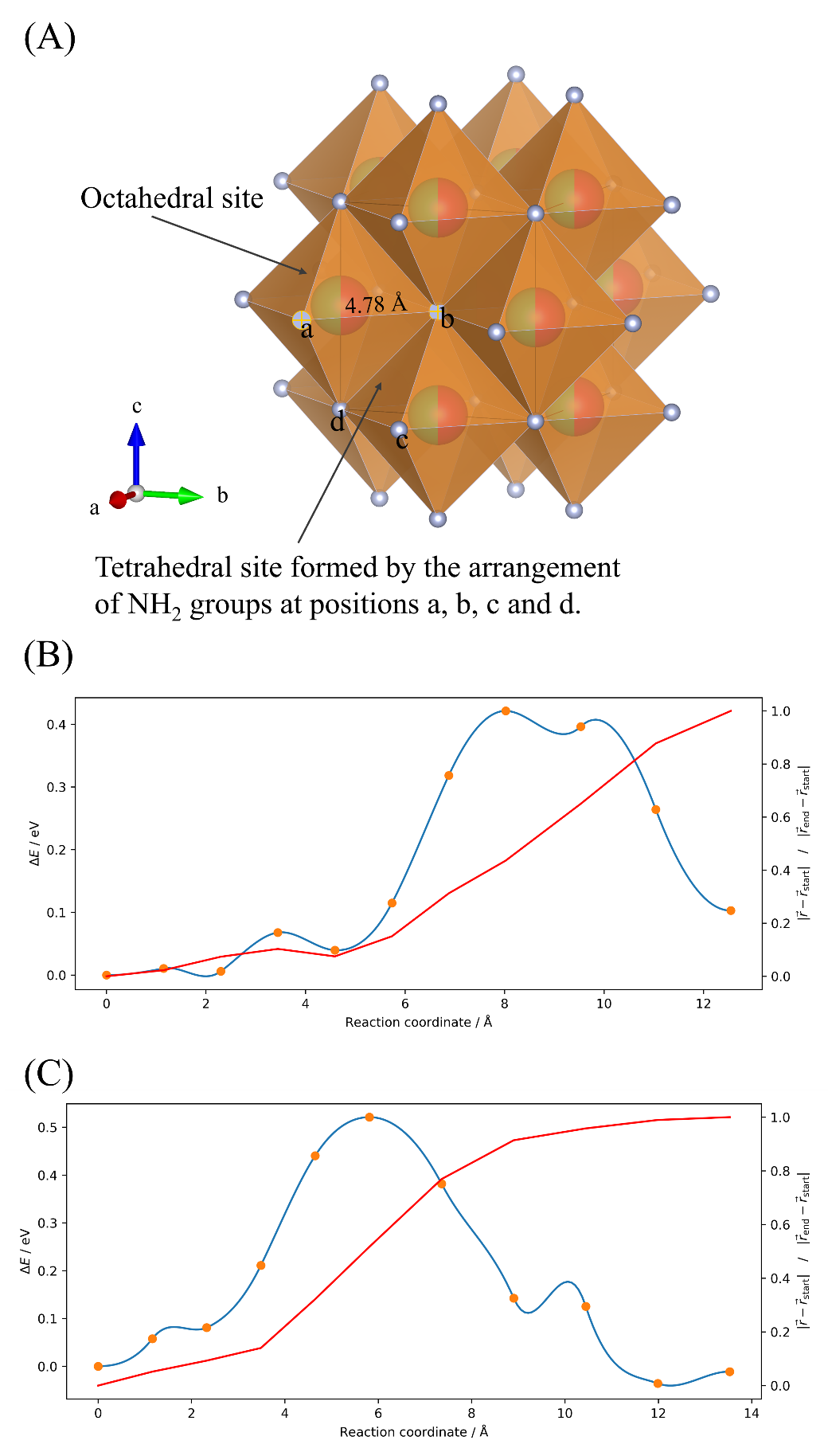


**Figure S5**. (**A**) Structure of Rb_0.5_Cs_0.5_NH_2_ solid solution showing the N-N bond distance of 4.78 Å. Cation migration energy profile for octahedral-to-octahedral paths in (**B**) RbNH_2_ and (**C**) CsNH_2_.

**Discussion of “paddlewheel effect” characteristics:**

The concept of the "*paddlewheel effect*", where anion rotations facilitate cation mobility, has been subject of considerable debate in the field of solid-state ionics ^[^[^23^](#_ENREF_23)^,^ [^25-29^](#_ENREF_25)^]^. In the following, we briefly present the current state of this on-going discussion by summarizing the arguments of two differing interpretations and subsequently show where we place our findings in this spectrum.​

A study by Jun et al. ^[^[^28^](#_ENREF_28)^]^ disputes the existence of a literal "*paddlewheel effect*", i.e. large-angle anion rotations assisting ion diffusion, after having performed AIMD simulations, stating that such rotations occur too rarely and with energy barriers too high to significantly impact ionic conductivity. Instead, their study proposes a "*soft-cradle mechanism*", where static tilting of isolated anion groups optimizes Li⁺ coordination environments, thus reducing migration barriers without requiring dynamic rotational coupling.

On the other side of the debate is a reply by Smith and Siegel ^[^[^29^](#_ENREF_29)^]^ to Jun et al.’s claims, that argues that the definition of the "paddlewheel effect" used by Jun et al. in their publication is overly restrictive and leads to an incorrect conclusion about its non-existence. By adopting a broader, rather physically motivated definition where anion rotations larger than liberations facilitate cation mobility, the authors demonstrate that Jun et al.'s own data, along with existing literature ^[^[^51^](#_ENREF_51)^,^ [^52^](#_ENREF_52)^]^, support the existence of the paddlewheel effect.

Considering the on-going debate, we chose to align ourselves more strongly with the broader definition by Smith and Siegel with the key aspects being:

- Anion rotations facilitate cation movement: Rotational motion of anion clusters can create transient pathways that assist in cation diffusion
- Presence of large rotations relative to liberations: Substantial rotational movements of anions are more effective in promoting cation mobility than smaller, oscillatory liberations
- Temporal and spatial correlation of reorientations: Anion reorientation and cation hopping should be correlated in both time and space, indicating a cooperative mechanism
- Maintaining local coordination: Anion orientations adjust in response to the movement of neighboring cations to preserve local coordination
- Comparable hopping and reorientation rates: The frequency of correlated cation hops and anion reorientations should be of similar within an order of magnitude

From our point of view, all these characteristics are met for the reaction pathway described in the main text and shown in the visualization (**Movie S1**) at time stamp 0:02-0:06. Here it is clearly visible in the bottom-right view, how the NH_2_^-^ anion rotates counter-clockwise to allow transition of Rb^+^/Cs^+^.

**References**

[

51. M. Jansen: 'Volume Effect or Paddle-Wheel Mechanism—Fast Alkali-Metal Ionic Conduction in Solids with Rotationally Disordered Complex Anions', *Angewandte Chemie International Edition in English*, 1991, **30**(12), 1547-1558.

52. J. G. Smith and D. J. Siegel: 'Ion Migration Mechanisms in the Sodium Sulfide Solid Electrolyte Na3–xSb1–xWxS4', *Chemistry of Materials*, 2022, **34**(9), 4166-4171.
